# Cavernous Hemangioma of the Skull and Meningioma: Association or Coincidence?

**DOI:** 10.1155/2015/716837

**Published:** 2015-04-16

**Authors:** M. Kilani, M. Darmoul, F. Hammedi, A. Ben Nsir, M. N. Hattab

**Affiliations:** ^1^Neurosurgery Department, Fattouma Bourguiba University Hospital, Medical University, Monastir, Tunisia; ^2^Pathology Department, Fattouma Bourguiba University Hospital, Medical University, Monastir, Tunisia

## Abstract

Intraosseous cavernous hemangiomas of the skull are rare. Meningiomas are quite frequently encountered in a neurosurgical practice. The association between these two entities is nevertheless very uncommon. The authors present a case of a 72-year-old woman suffering from headache. The MRI showed a parietal meningioma with adjacent thick bone. The meningioma and the bone were removed. The histological examination confirmed the diagnosis of meningioma and revealed a cavernoma of the skull. The relationship between the lesions suggests more than a coincidental association. Several hypotheses are proposed to explain common causal connections.

## 1. Introduction

Primary intraosseous cavernous hemangiomas (PICH) are benign tumors arising from intrinsic vasculature of the bone [1]. Histopathologically, intraosseous hemangiomas are classified as venous, cavernous, or capillary type according to their vascular network. They most commonly occur in spinal vertebral column. They are rarely seen in the calvarium and account for only 10% of all benign skull tumors [2].

Meningiomas are benign tumors frequently encountered in the neurosurgical practice.

The development of a calvarial cavernous hemangioma and a meningioma in the same region has never been reported to date.

We present a case in which this unusual association was found.

## 2. Case Report

### 2.1. History, Examination, and Neuroimaging Findings

**Figure 1:**
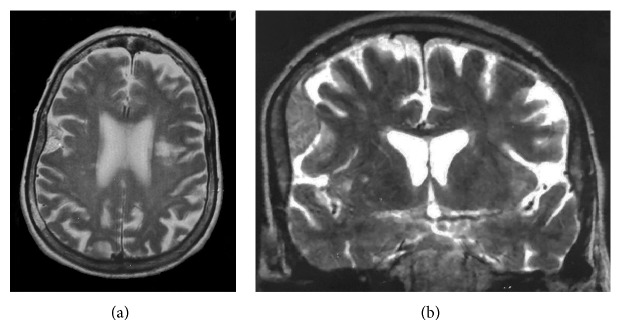
MRI images of the patient. Axial (a) and coronal (b) T2-weighted images show a hyperintense extra-axial right parietal convexity space occupying lesion, compatible with a meningioma. The skull adjacent to the lesion is thicker than contralateral bone. The separation plan is hypointense.

This 72-year-old woman presented at our department with headache without neurological disturbances. A plain and contrast enhanced MRI was performed. It showed a well-defined, extra-axial right parietal convexity space occupying lesion. The lesion was isointense on T1 weighted images and hyperintense on T2 weighted images. The lesion showed intense homogenous postcontrast enhancement. In view of these characteristic findings the lesion was diagnosed as a meningioma. The bone was larger than the contralateral side, but an intradiploic tumor was not suspected (Figure 1). Since it was a relatively small meningioma, neither angiography nor embolization was considered.

### 2.2. Operation and Postoperative Course

Surgical removal of the lesion via a right parietal approach was decided. On surgery the bone was thick with blue colored areas, so it was removed. No adherence to the dura mater was noticed. The tumor was actually extra-axial and arose from the dura mater. Moreover, the consistency of the lesion was that of a typical meningioma. After the removal of the meningioma, cranioplasty was performed by methylmethacrylate into the bone defect. At the first postoperative day, the patient developed fever 39°C, tachycardia with heart rate 100 bpm, and hypoxia with oxygen saturation at 85%. Laboratory parameters indicated respiratory acidosis. Chest X-ray was suggestive of bibasilar opacities. The patient was intubated and started on Vancomycine and Amikacine. Despite adequate measures, she succumbed to septic shock in less than 1 day from the time of clinical diagnosis. Blood culture reported growth of* Acinetobacter aeruginosa*.

### 2.3. Histopathological Findings

**Figure 2:**
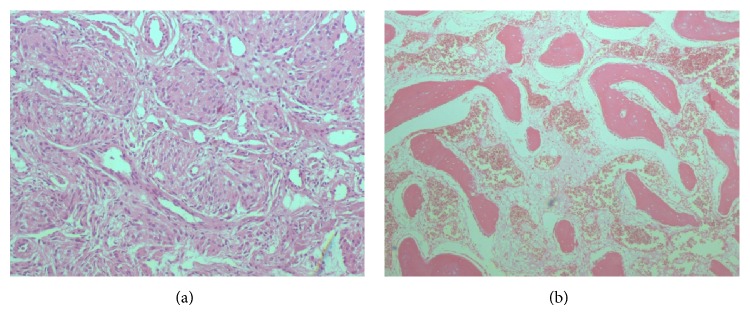
(a) Photomicrograph showing thin-walled vascular channels lined by thin layer of endothelial cells interspersed among bony trabeculae, compatible with cavernous angioma of the skull. Hematoxylin and eosin stain, ×4. (b) Nested aggregate of epithelioid cells, compatible with meningothelial meningioma. Hematoxylin and eosin stain, ×20.

Histologic examination of the bone revealed bone trabeculae widely separated by loose connective tissue enclosing multiple thin-walled vascular spaces lined by endothelial cells, suggesting cavernous hemangioma of the skull (Figure 2(a)). This exam confirmed also the diagnosis of a meningothelial meningioma (Figure 2(b)).

## 3. Discussion

Intraosseous cavernous hemangiomas are benign tumors of blood vessels, most commonly found in the spinal vertebral column followed by the skull [2]. They are mostly located in the frontal bone [3]. They are usually solitary [4]. These lesions are slowly growing, occurring in women in the fourth decade of life [5]. Neurological deficits are unusual since these tumors tend to expand externally [6]. The medical history is usually nonspecific. The major symptom is usually a visible or palpable bony hard mass, slowly growing covered by normal skin [1]. The treatment is complete tumor removal with normal bony margins [2].

Meningiomas arise from meningothelial cells of the arachnoid layer that forms the external lining of the brain and occur primarily at the base of the skull in the parasellar regions as well as over the cerebral convexities [7]. Meningiomas may be categorized as benign (90%), atypical/borderline (5%), and malignant (3–5%); within the benign category, there are several subtypes, including syncytial, fibrous, and transitional [8]. In general, these lesions are solitary (with the exception of individuals with neurofibromatosis for whom multiple lesions are common) and come to clinical attention as a result of symptoms including seizure, hemiparesis, or cranial neuropathy, generally associated with compression by the meningioma of surrounding neural tissues. The treatment of choice for meningioma is surgical excision. However, total resection is not always feasible especially in large meningiomas, high neurovascular risks, or medically unfit patients [8].

Very few reports mention the coexistence of vascular lesions and meningioma in the same patient [7–10]. To our knowledge, we report the first case of a calvarial cavernous hemangioma located nearby a meningioma.

Cavernous hemangiomas can be misdiagnosed as a meningioma, especially when localized in the cavernous sinus [11]. Diagnosing cavernous sinus CH preoperatively is very important, but its radiological differential diagnosis is quite difficult. Cavernous sinus CHs are usually misdiagnosed as a meningioma because meningioma is a common lesion. Since CHs are rich in vascular structures and near the important neurovascular structures, its removal by surgery both is difficult and has serious complications [11].

Initially the thickened calvarium was thought to be hyperostosis. Bony hyperostosis is a common sign of meningioma, in which the hyperostotic bone is usually smaller than that of the underlying tumor. Generally, the histopathological exam reveals the infiltration of the tumor between bony trabeculae [12], which was absent in this case. Furthermore the thickened skull was larger than the tumor. In the imaging, we can see easily a separation plan between the two entities confirming the individuality of each lesion. Although the cavernous hemangioma was not well identified in the imaging MR, it was confirmed by the histopathological exam. We think it is due to the precocity of the diagnosis of the tow lesions (small meningioma).

The second question raised by the present report deals with the hypotheses of this association. An environmental factor causing both lesions can be speculated.

Since the time of Harvey Cushing (24 cases of operated meningiomas developed in the site of a previous trauma) [13], head trauma has been suggested as a risk factor for meningioma, although the results across studies are not consistent. Several small case control studies from the early 1980s report an increased risk of meningioma associated with head trauma for both males and females [8].

Previous trauma has been anamnestically found in 25% of frontal bone hemangiomas. Trauma mechanisms involved falling on the head or a blow to the head with a stick. The hemangioma developed exactly on the site of the preceding trauma, as has been documented radiologically [3]. A causal relationship has been even suggested [3].

Exposure to ionizing radiation has been implicated as an etiology for brain tumors like meningiomas and cavernous hemangiomas, gliomas, and sarcomas [7]. Radiation-induced neoplasms often develop as a result of mutagenic capacity, chromosome aberration, and DNA injury caused by the radiation. Lesions associated with this exposure may be multiple and may be associated with high recurrence rates [8]. Data from atomic bomb survivors exposed to high doses show a greatly increased risk for meningioma [8]. Evidence also exists for lower dose levels. Radiation-induced meningiomas have a tendency to be more aggressive compared to those de novo meningiomas. There is a slight male preponderance.

Postradiation cavernous angiomas have certain special features. These have a higher tendency for clinical overt bleeding [14]. The development of both meningiomas and cavernous angioma following prophylactic cranial irradiation as a part of treatment of acute lymphoblastic leukemia has been reported [7]. It is worth mentioning that our patient has never been irradiated.

Although the implication of ionizing radiation and head trauma in the development of meningiomas and cavernous hemangiomas is well documented, we report the first case of an association meningioma-calvarial cavernoma with no etiology.

## 4. Conclusion

We present a unique case of intraosseous cavernous hemangioma associated with a meningioma. It is a very uncommon case for many reasons: the rarity of calvarial hemangioma, the unusual association hemangioma-meningioma, and their development in the same location.

The occurrence of these coexisting lesions could be coincidental but may be explicable on the basis of common inducing factors.
